# Mild chronic perturbation of inhibition severely alters hippocampal function

**DOI:** 10.1038/s41598-019-52851-w

**Published:** 2019-11-11

**Authors:** Min-Yu Sun, Luke Ziolkowski, Peter Lambert, Hong-Jin Shu, Micah Keiser, Nicholas Rensing, Natasha Warikoo, Monika Martinek, Carson Platnick, Ann Benz, John Bracamontes, Gustav Akk, Joe Henry Steinbach, Charles F. Zorumski, Michael Wong, Steven Mennerick

**Affiliations:** 1 Department of Psychiatry, Washington University School of Medicine, St. Louis, USA; 2 Department of Neurology, Washington University School of Medicine, St. Louis, USA; 3 Department of Anesthesiology, Washington University School of Medicine, St. Louis, USA; 4 Department of Neuroscience, Washington University School of Medicine, St. Louis, USA; 5 Taylor Family Institute for Innovative Psychiatric Research, St. Louis, USA; 6 MSTP Training Program, Washington University School of Medicine, St. Louis, USA

**Keywords:** Ion channels in the nervous system, Inhibition-excitation balance, Encephalopathy

## Abstract

Pentameric GABA_A_ receptors mediate a large share of CNS inhibition. The γ2 subunit is a typical constituent. At least 11 mutations in the γ2 subunit cause human epilepsies, making the role of γ2-containing receptors in brain function of keen basic and translational interest. How small changes to inhibition may cause brain abnormalities, including seizure disorders, is unclear. In mice, we perturbed fast inhibition with a point mutation T272Y (T6′Y in the second membrane-spanning domain) to the γ2 subunit. The mutation imparts resistance to the GABA_A_ receptor antagonist picrotoxin, allowing verification of mutant subunit incorporation. We confirmed picrotoxin resistance and biophysical properties in recombinant receptors. T6′Y γ2-containing receptors also exhibited faster deactivation but unaltered steady-state properties. Adult T6′Y knockin mice exhibited myoclonic seizures and abnormal cortical EEG, including abnormal hippocampal-associated theta oscillations. In hippocampal slices, picrotoxin-insensitive inhibitory synaptic currents exhibited fast decay. Excitatory/inhibitory balance was elevated by an amount expected from the IPSC alteration. Partial pharmacological correction of γ2-mediated IPSCs with diazepam restored total EEG power toward baseline, but had little effect on the abnormal low-frequency peak in the EEG. The results suggest that at least part of the abnormality in brain function arises from the acute effects of truncated inhibition.

## Introduction

GABA is the major inhibitory transmitter in the brain and sculpts activity throughout the lifespan in large part by activating ionotropic GABA_A_ receptors (GABA_A_Rs). Synaptic GABA_A_Rs typically contain a γ subunit, and γ2 expression level is more abundant than γ1^1^ or γ3.The γ2 subunit is important for pharmacological and biophysical properties of GABA_A_ receptors, as well as synaptic receptor localization^2–6^. Mutations in γ2 may help us understand the role of inhibition in brain functioning and dysfunction. Indeed, several mutations in γ2 result in human epilepsy syndromes^7,8^.

γ2 is a non-obligatory subunit (unnecessary for ligand binding or channel gating), but its presence reduces channel deactivation rate^6^. It is also important for proper targeting of GABA_A_ receptors^9^. Complete loss of the γ2 subunit in mice is lethal, and heterozygote null mice display altered synaptic receptor localization and an anxious/depressed behavioral phenotype^10,11^. The human K328M γ2 mutation, in the M3-M4 extracellular loop of the subunit, speeds channel deactivation kinetics without altering steady-state or pharmacological properties of GABA_A_ receptors^12^. These previous observations make γ2 mutations, short of deletion, an attractive target to perturb inhibition.

We studied the impact of altered inhibition by engineering a point mutation in the M2 domain of the endogenous mouse γ2 subunit (T6′Y mutation). The mutation we adopted reduces picrotoxin sensitivity of the receptor without changing sensitivity to GABA^13–15^. Picrotoxin insensitivity provides a pharmacological signature, allowing verification that the mutated γ2 subunit (γ2*) subunit is incorporated and trafficking to synaptic sites. We found that recombinant receptors containing the γ2*subunit also exhibit accelerated GABA_A_ deactivation kinetics, similar to a human mutation (K328M), thereby providing a route for controlled and translationally relevant disruption of phasic inhibition.

Our results show that mice homozygous for the γ2*subunit exhibit disrupted hippocampal function, measured with EEG and by c-Fos immunoreactivity. We explored underpinnings of the hippocampal disruption in brain slices and observed the expected acceleration of IPSC decay. The disruption altered the balance between spontaneous excitation and spontaneous inhibition in CA1 of hippocampus to a degree expected of the mild receptor phenotype. Diazepam treatment rescued IPSC kinetics to WT status, but treatment of mutant mice at a non-sedative dose only partly restored EEG properties toward the WT profile. We hypothesize that altered hippocampal function in adulthood arises from a combination of the primary genetic lesion and additional secondary consequences, including potential compensatory alterations, of the mutation that remain to be defined. The mouse model thus serves as a tool for exploring the consequences of mildly perturbed inhibition.

## Methods

### Ethical approval

All animal procedures were performed according to NIH guidelines and approved by the Washington University Institutional Animal Care and Use Committee, protocol 20180208. Pain and suffering was alleviated with appropriate anesthesia and analgesia during tissue harvest and surgical procedures. Animals were derived in house as described below and were reared under the care of the Washington University School of Medicine Division of Comparative Medicine. Animals had ad libitum access to food and water throughout. Mice were euthanized at the end of studies according to NIH guidelines for minimizing pain. Other aspects of animal studies are described in the sections below.

### Knock-in mice

γ* knock-in (KI) mice were generated using CRISPR/Cas9-based genome editing, as described previously^16^. Briefly, Cas9 mRNA, sgRNA, and ssODN constructs were injected into the cytoplasm and pronucleus of fertilized eggs. Injected eggs were cultured at 37 °C under 5% CO_2_ overnight, after which 20–25 two-cell stage embryos were transferred into oviducts of pseudopregnant females. The mutant allele of the γ* KI mouse was confirmed by PCR based genotyping from tail DNA.

Mosaic founders were bred with WT mice. We screened and identified F1 mice with the desired mutations to establish colonies by heterozygote crosses. Littermate WT controls, variably obtained from the γ* KI colony, were used for all experiments. Mice were maintained on a mixed C57BL/6CBA background. To confirm that the strategy did not result in unintended mutations of the CRISPR/Cas9 approach, we sequenced the entire γ2* gene, as well as the top 10 alternative genes likely to be targeted by the guide RNAs in three homozygous WT mice and three homozygous γ2* mice. We found no evidence for off-target mutations in these assays.

### Slice preparation

Hippocampal slices were prepared from postnatal day 34 (P34) to P57 GABA_A_R γ* KI or WT littermates of both sexes. The ages avoided developmental changes in IPSC kinetics, which stabilize after P21^17^. We used mice of both sexes. Numbers of males and females were approximately balanced in each experiment.

In accordance with protocols approved by the Washington University IACUC, mice were anesthetized deeply with isoflurane and decapitated. The brain was removed and glued onto a Leica VT1200 specimen holder. Sagittal (300–400 μm) slices were cut in ice-cold, modified artificial cerebrospinal fluid (ACSF) (in mM: 87 NaCl, 75 sucrose, 25 glucose, 25 NaHCO_3_, 2.5 KCl, 1.25 NaH_2_PO_4_, equilibrated with 95% oxygen −5% CO_2_ plus 0.5 CaCl_2_, 3 MgCl_2_; 320 mosmol). Slices were then incubated at 32–34 °C for 30 min in choline-based ACSF (in mM: 92 choline chloride, 25 glucose, 30 NaHCO_3_, 2.5 KCl, 1.2 NaH_2_PO_4_, 20 HEPES, 2 thiourea, 5 Na ascorbate, 3 Na pyruvate, 2 CaCl_2_ and 1 MgCl_2_, equilibrated with 95% oxygen-5% CO_2_; 300 mosmol), and subsequently stored at room temperature in regular ACSF (in mM: 125 NaCl, 25 glucose, 25 NaHCO_3_, 2.5 KCl, 1.25 NaH_2_PO_4_, equilibrated with 95% oxygen-5% CO_2_ plus 2.6 CaCl_2_, 1.2 MgCl_2_; 310 mosmol), allowing for at least one hour recovery prior to experiments. Except for noted exceptions, drugs were obtained from Sigma (St. Louis, MO).

### Whole-cell patch-clamp recording in slices

Slices were transferred to a recording chamber and continuously perfused with oxygenated, regular ACSF at 2 ml min^−1^. Experiments were performed at 30–32 °C. Somatic, whole-cell patch-clamp recordings were performed using differential contrast interference microscopy under infrared illumination. Dentate granule cells (DGCs) or CA1 cells were identified on an upright Nikon Eclipse E600FN microscope using a QImaging camera controlled with QCapture (QImaging, Surrey, Canada). Somatic whole cell recordings were made with borosilicate patch pipettes (World Precision Instruments, Sarasota, FL; Sutter Instruments, Novato, CA), having open tip resistance of 3–7 MΩ. After a whole-cell configuration was established, cells were allowed to fill with the intracellular solution for ∼5 min. Recordings were obtained using a MultiClamp 700B amplifier (Molecular Devices; Sunnyvale, CA), Digidata1550 16-bit A/D converter, and pClamp 10.4 software (Molecular Devices). Pipette capacitance was compensated using MultiClamp 700B Commander software. Somatic access resistance values were between 10–25 MΩ, and cells with unstable access resistance (>20% change) were excluded from analysis.

### Measurement of spontaneous EPSCs and IPSCs

For calculating the ratio of spontaneous EPSCs (sEPSCs) and IPSCs (sIPSCs), cells were held at −60 mV and 0 mV, respectively, with an intracellular pipette solution containing (in mM): 120 cesium methanesulfonate, 20 HEPES, 10 EGTA, 2 MgATP, 0.3 Na2GTP, and 5 QX-314; pH was adjusted with CsOH to pH 7.25 (290 mosmol). No receptor antagonists were present for this experiment. To verify diazepam’s effects on GABA_A_ responses, pharmacologically isolated sIPSCs were recorded at −70 mV with an intracellular pipette solution containing (in mM): 130 CsCl, 10 HEPES, 5 EGTA, 2 MgATP, 0.5 NaGTP, and 4 QX-314; pH was adjusted with CsOH to pH 7.3; 290 mosomol. GABA_A_R sIPSCs were isolated by blocking ionotropic glutamate receptors with 10 μM NBQX (Tocris, Bristol, United Kingdom) and 50 μM D-APV (Tocris). All the sEPSC and sIPSC data were acquired in gap-free mode at 5 kHz, filtered at 2 kHz using an 8-pole Bessel filter.

### CA1 Field EPSP (fEPSP) recording

Extracellular field excitatory postsynaptic potentials (fEPSPs) were recorded in CA1 stratum radiatum 50 μm below the pyramidal cell layer with glass electrodes (1 MΩ) filled with ACSF. Stimuli were delivered to the commissural/Schaffer collateral afferents with a concentric bipolar electrode positioned parallel to the recording electrode ~500 μm away. Input/output curves were generated by incrementing the stimulation amplitude from 50 to 600 μA. The standard stimulation intensity for trains was set to an intensity that evoked 50–75% of the maximal fEPSP amplitude. A 50 Hz, 5-pulse train was then applied every 20 s for 20 times, and data were analyzed from averaging the 20 sweeps. fEPSP slope was measured from ~20–80% of the peak.

### Sholl analysis

DGCs were patched and infused with CsCl-based internal solution containing 0.1% Lucifer Yellow CH dilithium salt (Sigma L0259). After staining for 10–15 minutes, the pipette was withdrawn from the cell, and the slice was fixed in 4% PFA for 10 minutes, followed by 3 washes with PBS. The slice was then incubated in blocking solution containing 5% sucrose, 2% BSA, and 1% Triton X-100 in PBS for 1 hr. Next, the slice was incubated with anti-Lucifer Yellow rabbit primary antibody (1:2000, Thermo Fisher Scientific Cat# A-5750, RRID:AB_2536190) diluted in blocking solution for 2 hr. After rinsing 3 times with PBS, the slice was incubated in a goat anti-rabbit secondary antibody (Alexa Fluor 488, 1:200, Invitrogen) diluted in PBS for 1 hr. All incubations were at room temperature. The slice was then mounted on a glass slide.

Following immunofluorescence staining, the Lucifer Yellow-filled cells were scanned using a Nikon C1 confocal attachment on a Nikon TE2000 inverted microscope platform with a 60X objective. Z-series stacks were taken to include the entire dendritic arbor of each cell (around 60 μm each) with a thickness of around 1 μm. Multiple (2–3) z-series were taken in order to fully capture each cell, and the cells were reconstructed from the z-series using the pairwise stitching function in ImageJ. Dendritic arbors were skeletonized using the Simple Neurite Tracer plugin^18^, and analyzed using the Sholl Analysis plugin with the soma as the center point and 10 μm intervals between concentric circles^19^.

### Other immunostaining

Other primary antibodies used include rabbit anti-c-Fos (1:1000; Millipore Cat# ABE457, RRID:AB_2631318, rabbit anti-somatostatin (1:1000; Peninsula Laboratories Cat# T-4103.0050, RRID:AB_518614), and rabbit anti-parvalbumin (1:1000; Swant Cat# PV-28, RRID:AB_2315235). In each case visualization was achieved as described above.

### N2A cell transfection

Murine neuro-2a (N2a; ATCC Cat# CCL-131, RRID:CVCL_0470) cells were cultured in DMEM with 10% FBS, 2 mM glutamine plus 100 U/ml penicillin, and 0.1 mg/ml streptomycin in 5% CO_2_ and 95% air. Cells were transiently transfected with α1, β2, and either WT γ2 or edited γ2* subunit plasmid DNA during 4 hour incubation in serum-free medium and Lipofectamine 2000 reagent (Invitrogen) at 37 °C.

### Culture electrophysiology

Whole-cell and cell-attached patch-clamp recordings of transfected N2a cells were obtained on an Eclipse TE2000-S inverted microscope using a MultiClamp 700B amplifier, Digidata 1550 16-bit A/D converter and pClamp 10.4 for data collection. Whole-cell recordings were performed with borosilicate patch pipettes of 3–7 MΩ containing (in mM): 130 CsCl, 4 NaCL, 10 HEPES, 5 EGTA, and 0.5 CaCl_2_. Extracellular solution typically contained (in mM): 138 NaCl, 4 KCl, 10 HEPES, 2 CaCl_2_, 1 MgCl_2_ and 10 glucose. Some recordings of reversal potential were obtained with methanesulfonate substitution for chloride. Cells were voltage clamped at −70 mV for whole-cell recordings unless otherwise indicated. For cell-attached single channel recordings, patch pipettes contained 50 µM GABA in the described extracellular solution and cells were voltage clamped at approximately −60 mV. Data were sampled at 50 kHz and filtered at 2 kHz. Clusters of channel openings were isolated for analysis as described^20^. For experiments using local drug delivery, solutions were flowed onto cells using a gravity-driven perfusion system with exchange times of ~100 ms. Fast application (~1 ms exchange times) of solution was achieved by rapidly switching between two solution flows from a theta-pipette attached to a piezo-electric actuator. Junction currents obtained following patch blow-out are shown above traces to indicate drug delivery and removal speed.

### EEG surgery and recording

Methods were as previously described^21^. In brief, mice of both sexes were anesthetized with isoflurane (5% for induction, 1.5–2% for surgery), and mounted in a stereotactic frame (Kopf, Tujunga, CA). Bilateral holes were drilled in the skull for attachment of epidural EEG screw electrodes, a reference, and a ground. For depth recordings, wires were inserted into the hippocampus, and sites were verified upon euthanasia of the animals at the end of recording. Animals recovered for 3 days before initiating video EEG monitoring.

### Statistics

For sEPSCs and sIPSCs, events were detected using a template-matching algorithm in Clampfit (Clements and Bekkers, 1997); templates were created by averaging >20 events. For quantification of total charge, Clampfit statistics for area measurement was used on the extracted events after subtraction of baseline current. Decay times were assigned based on single or bi-exponential least-squares fits as appropriate. For bi-exponential fits, we collapsed the two components into an overall weighted decay time constant for pooling and for comparisons. The weighted time constant was calculated as ΣA_i_*τ_i_, where A_i_ is the fractional amplitude and τ_i_ is the time constant of the component. For determining the effects of 5 Hz, 5-pulse stimulus trains on CA1 fEPSPs, 20 trials were averaged, and the 2nd-5th test fEPSP slopes were normalized to the slope of the initial average conditioning fEPSP slope. Single-channel data were analyzed by modeling channel open and closed states in QuB software.

To generate EEG power spectra, vigilance states were scored manually in 15 second epochs as either “Active” or “Inactive” during the animal’s dark cycle using Stellate Reviewer software. For active segments, our analysis criteria required movement for a (1–5 min) block, within which periods of inactivity were no more than 15 s each. Multiple active segments were concatenated for analysis. For diazepam experiments, segments were taken from all periods of activity during the hour immediately following the injection. We manually rejected segments containing >1 Hz voltage spikes of >1000 μV or containing signal dropout. Data were collected at a rate of 200 Hz, and bandpass filtered at 1–100 Hz with a digital notch filter at 60 Hz. Selected active periods were exported to Clampfit, where power spectra were calculated by fast Fourier transform with a Von Hann window, using the average of 512 spectral segments with 50% window overlap. The mean power spectrum for the two hemispheres was calculated to represent each animal. Power in each frequency bin was then normalized to the total area of the spectrum (1–100 Hz). Frequency peaks were found using a Matlab script that was set to find all local maxima that were larger than the surrounding data points by a value of at least 1/24 the range of amplitude values. Frequency bands were defined as follows (in Hz): δ 1–5, θ 5–9, α 9–12, β 12–30, γ 30–100. Power from frequencies between 55–65 Hz was excluded from the analysis of the power spectra.

Cells or patches served as the statistical basis of N for all electrophysiology and morphology experiments; animal number is reported in figure legends. Student’s independent, two-tailed t-tests were performed to determine statistical significance between the means of two groups. For detecting interaction between two genotypes at multiple times, two-way ANOVA and Bonferroni corrected, post hoc analyses were performed. For immunostaining results, stains were performed in paired rounds of matched WT and γ2* animals. Because age, litter, time of day, staining reagents, etc. were matched for each round of staining, we employed a dependent sample design^22^. For multiple group (>2 groups) comparisons, one way ANOVA and Bonferroni corrected, post hoc analyses were performed. For comparisons of EEG bands with multiple t tests, false discovery rate was controlled by a two-stage linear step-up procedure of Benjamini, Krieiger, and Yekutielli, with the desired false discovery rate set at 1%. For detection of effects within cells, a paired t-test was performed. Statistical analysis was performed using GraphPad Prism 7 or 8 (GraphPad Software, San Diego, California). For clarity, specific tests are described in the Results and figure legends. In figures significance is displayed at the level of *p ≤ 0.05, **0.01, and ***0.001. Actual P values are given where practical. Summary data are presented as mean ± standard error of the mean (SEM). All original data are available by request from the authors.

## Results

**Figure 1:**
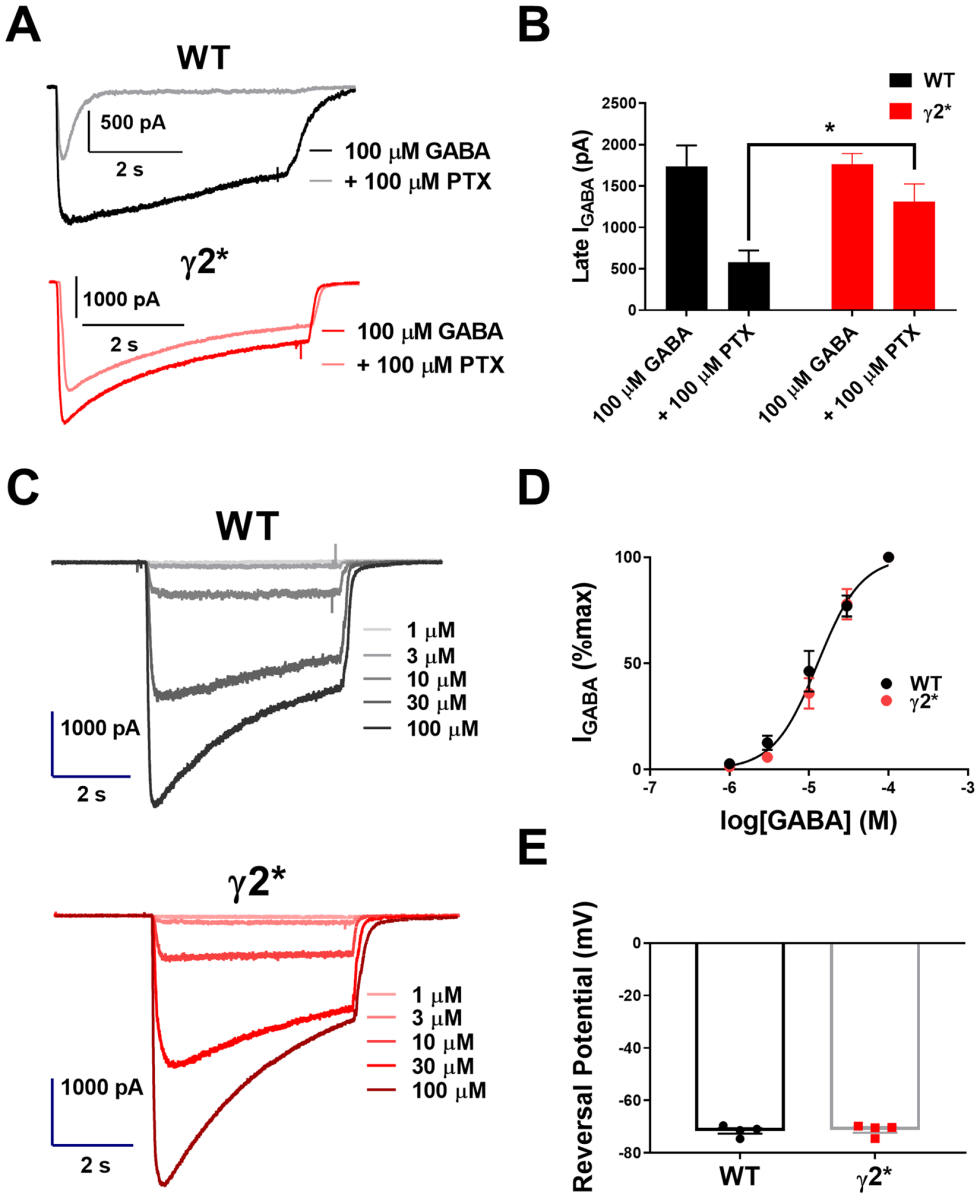
GABA responses from N2a cells expressing WT γ2 or γ2*, with WT α1 and β2. (**A**) GABA responses (100 μM) ± 100 µM PTX from representative WT and γ2* expressing cells. (**B**) Summary of 8 cells. *P = 0.013 by Welch’s t test. (**C**) Responses from increasing concentrations of GABA (1, 3, 10, 30, and 100 μM) from representative WT and γ2* expressing cells in the absence of PTX (N2a cells). (**D**) Dose-response curves of WT (n = 9) and γ2* (n = 10) expressing cells, fit with the Hill equation and yielding similar EC50 values (see text). (**E**) Reversal potential of WT and γ2* receptors obtained with a cesium methanesulfonate solution as detailed in the Methods.

Consistent with previous results, we confirmed with recombinant receptor subunits, expressed in N2a cells, that γ2* subunit-containing receptors, transfected with WT α1 and β2 subunits, imparted picrotoxin resistance and failed to alter GABA concentration-response characteristics (Fig. 1A–D)^13^. The response of WT receptors to picrotoxin exhibited the appearance of a rapidly desensitizing current (Fig. 1A), consistent with previously documented activation dependence of picrotoxin inhibition^23,24^. The GABA EC50 values for WT and mutant receptors did not differ statistically at 11.5 and 14.1 μM respectively. Macroscopic desensitization percentage with 5 s at 100 μM GABA also did not detectably differ (53 ± 6% WT vs. 45 ± 4% γ2*, P = 0.29). Reversal potential of currents with a low [Cl^−^] pipette solution was indistinguishable from WT receptors (Fig. 1E), suggesting similar Cl^−^ permeability.

**Figure 2:**
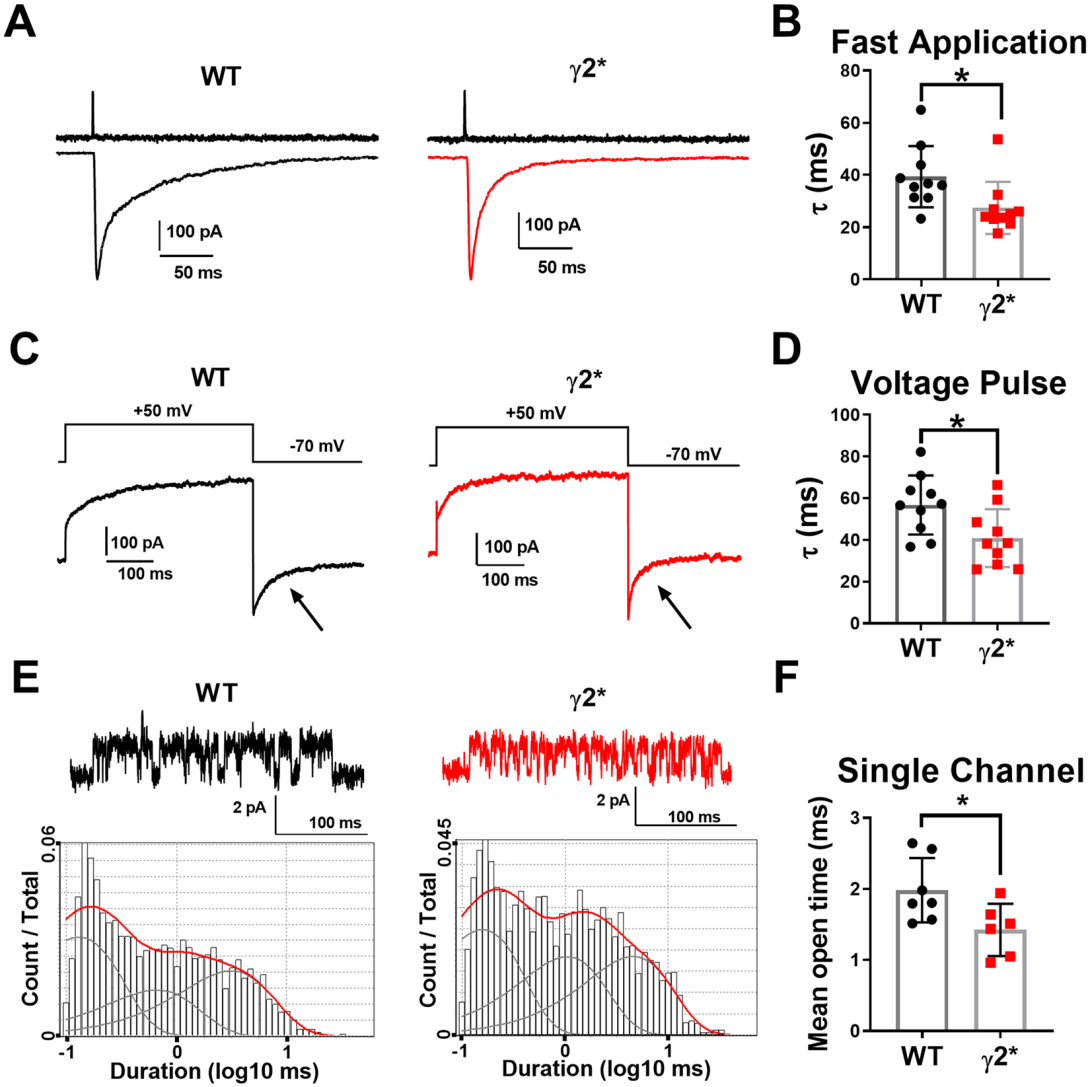
Faster γ2* deactivation. (**A**) Response of nucleated patches from N2a cells to 1 ms application of GABA. Upper trace: open tip current showing application speed. (**B**) Summary. *P = 0.025, two-tailed t test. (**C**) Voltage-pulse relaxation test of deactivation kinetics. * P Non-GABA currents are subtracted. (**D**) Tau of tail currents measured at −70 mV (arrows in **C**). *P = 0.021, two-tailed t test. (**E**) Sample single-channel records from a cell-attached patch on N2a cells expressing the indicated subunits. Open state is up. The lower panels show log-binned counts of open times from samples of the two subunit variants. The fits are to three components. (**F**) Average weighted mean open time for each patch. *P = 0.035 two-tailed t test.

On the other hand, γ2* receptors in excised outside-out patches responded to synaptic-like, 1 ms pulses of 100 μM GABA with faster deactivation than receptors containing WT γ2 (Fig. 2A,B). We confirmed the altered kinetics with two additional, independent approaches. First, we exploited the voltage dependence of receptor gating at low [GABA]^25^. This approach originates from the finding, first made with nicotinic acetylcholine receptors, that voltage-pulse relaxations can be used to monitor changes in channel gating^26^. At 2 μM, leak-subtracted GABA-induced currents exhibited a time-dependent growth at +50 mV (Fig. 2C,D), reflecting voltage dependent increase in channel open probability^26^. Upon return to −70 mV, the current relaxed back to the original steady state value with an exponential time constant (Fig. 2C,D). This relaxation was faster in mutant receptors, confirming a kinetic difference between γ2* and WT γ2 receptors. This experiment employed a low GABA concentration because at high GABA concentration, the voltage dependence of GABA_A_R gating disappears^27^.

Second, we examined behavior of single channels gated by 50 µM GABA in cell-attached patches obtained from N2a cells. We used a high concentration of GABA to promote long-lived desensitized states, thereby promoting separation of bursts of channel activity^20^. Here we analyzed channel open times and closed times in bursts of channel activity, as previously described^20^. We found that γ2* channels exhibited lower mean open times than WT γ2 channels (Fig. 2E,F), offering a substrate for the altered deactivation kinetics observed in experiments on macroscopic currents. The same data set yielded no evidence for a difference in channel closed times (WT, 1.7 ± 0.2 ms vs. γ2*, 2.1 ± 0.4 ms, P = 0.4 Student’s t test).

**Figure 3:**
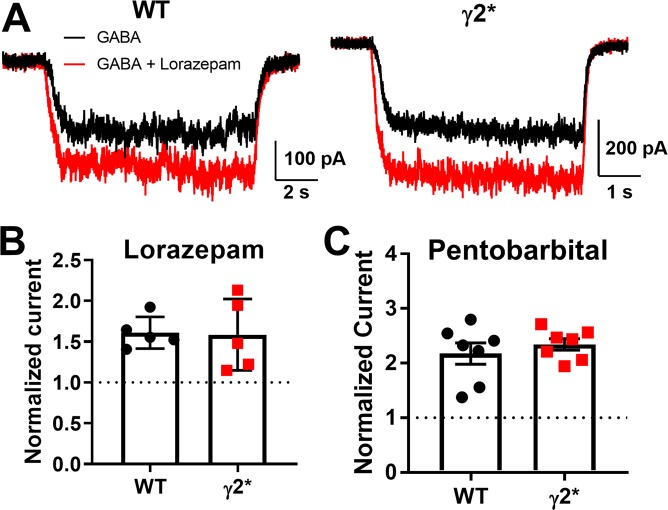
Allosteric potentiation intact in γ2*. (**A**) Sample traces from transfected N2a cells with WT α1 and β2 subunits, with the indicated γ2 subunit: 1 μM lorazepam at 2 μM GABA. (**B**,**C**) Summary of the effects of lorazepam and 50 µM pentobarbital, at 2 μM GABA. P = 0.912 lorazepam and P = 0.470, Student’s unpaired t tests.

Amino acid residues in the γ2 subunit, along with α subunit residues, are critical for potentiation by benzodiazepines^28,29^. Although the site for benzodiazepine binding is distant from the T6′Y mutation, long-range interference could be possible. Lorazepam (1 µM) evoked similar potentiation from N2a cells expressing γ2 and γ2* receptors (Fig. 3A,B). Similarly, a broad-spectrum (subunit-independent) potentiator, pentobarbital, also retained activity at γ2* receptors similar to WT γ2 receptors (Fig. 3C). These results suggest that receptors containing the mutated γ2* subunit maintain normal pharmacological responsiveness.

These results demonstrate that γ2* may be a tool to selectively perturb phasic inhibition *in vivo*. The observed changes to receptor function phenocopy properties of the K328M human epilepsy mutation, as documented in previous studies of heterologous cells and cultured rodent neurons^12,30^. The picrotoxin resistance in the γ2* mutation allows verification of subunit incorporation in a manner not possible with the human mutation. The selective changes to non-equilibrium responses mean that tonic currents mediated by γ2* should not be disturbed. Thus, to study the impact of the perturbation to phasic inhibition, we made a mouse line in which the endogenous γ2 gene was edited with the T6′Y mutation and became picrotoxin resistant (γ2*).

**Figure 4:**
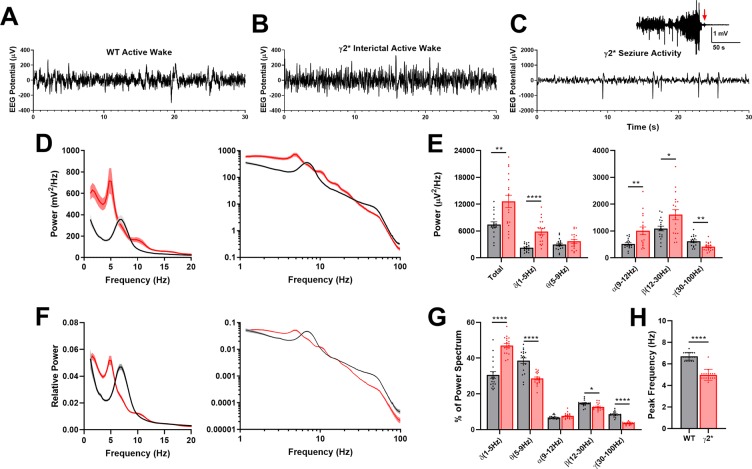
Cortical EEG abnormalities in γ2* mice. (**A**–**C**) Sample cortical EEG traces from WT and γ2* animals. The spikes in panel C were associated with myoclonic jerks. The inset shows brain activity preceding death in one γ2* animal. (**D**) Mean active wake power spectra from WT (Black n = 18) and γ2* (Red n = 18) animals Shading represents standard error. (**E**) Power of total spectrum and frequency bands shown in (**D**) (unpaired, corrected Student’s t tests, adjusted for false discovery, with asterisks indicating p value levels as given in the Methods). (**F**) Active wake power spectra normalized to total power of spectrum. (**G**) Frequency distribution of relative power (two-way ANOVA Genotype X Frequency Band shows significant interaction, (F (4, 136) = 45.90; P < 10^−15^). Results of individual corrected t tests are shown. (**H**) Peak location in the low frequency bands of the active wake power spectrum (unpaired Student’s t-test, P < 1e-4).

Mice homozygous for γ2* seemed to develop normally early, but tended to die prematurely. In a representative three month span out of 26 homozygote knock-in births, 11 animals died in their home cages before they were able to be used for experiments at an average age of 87.4 days. This never occurred with WT littermates. Prior to death, γ2* mice were of normal weight (22.6 g vs. 21.0 g for WT littermates at P50; P = 0.332). However, mutant mice exhibited occasional behavioral myoclonic jerks associated with spikes in cortical EEG (Fig. 4A–C). Death appeared to be associated with a large generalized seizure, confirmed behaviorally by video (Fig. 4C, inset). Heterozygotes displayed no obvious behavioral or lifespan differences from WT. To probe for brain abnormalities, we analyzed cortical EEG recordings during active wake periods from WT and γ2* littermates at P40–60. During active exploration, power spectra from WT animals exhibited strong power in theta (6–9 Hz) frequencies, as expected^31,32^ (Fig. 4). γ2* Littermates demonstrated higher power than WT at frequencies up to 20 Hz. In the gamma frequency range, power was reduced in γ2* animals (Fig. 4D,E). Aside from these overall amplitude changes, the prominent theta peak associated with active exploration in WT animals was not present in γ2* animals and appeared to shift into the delta (3–5 Hz) frequency range (Fig. 4D,F).

**Figure 5:**
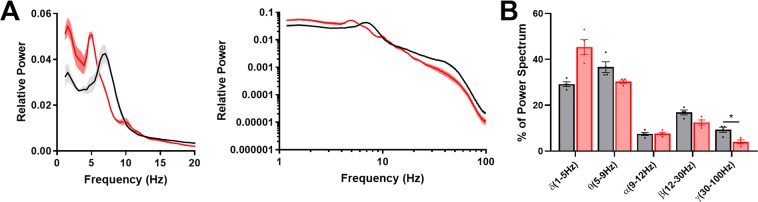
Hippocampal depth recordings recapitulate the EEG abnormality. (**A**) Normalized active wake power spectra from hippocampal depth electrodes (WT-Black γ2*-Red, n = 4 animals). (**B**) Frequency distribution of relative power (two-way ANOVA Genotype X Frequency showed significant interaction. F(4,24) = 15.50, P = 2e-6).

To confirm the changes in theta during active wake are evident in hippocampus, we performed depth EEG recordings from hippocampus. These recordings showed a very similar power spectrum during active wake as cortical EEGs (Fig. 5) and confirmed abnormal hippocampal function.

**Figure 6:**
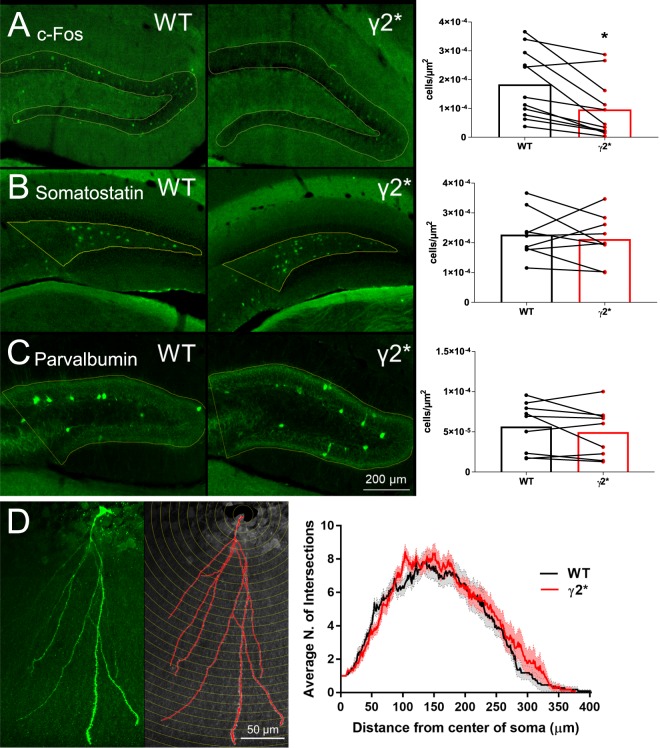
Depressed c-Fos immunolabeling in γ2* dentate in the absence of altered interneuron density or DGC morphological abnormalities. (**A**) c-Fos-positive DGC density in WT and γ2* KI slices. (N = 10 littermates per genotype, ages 31–100 days, P = *0.012; two-tailed paired t-test). (**B**) Somatostatin-positive cell density in the hilus of WT and γ2* KI slices. (N = 10 animals, P = 0.579; two-tailed paired t-test). (**C**) Parvalbumin-positive cell density in the dentate gyrus of WT and γ2* KI slices.KI slices. (n = 10 animals, P = 0.267; two-tailed paired t-test). Connected symbols in the graphs represent biological replicates of littermate pairs stained as a pair. (**D**) Representative confocal image of a Lucifer Yellow-filled DGC and subsequent Sholl Analysis comparing dendrite complexity of WT and γ2* KI DGCs. (n = 11 WT cells from 8 animals and 12 γ2* KI cells from 6 animals; P > 0.999; two-way repeated measures ANOVA).

We further explored hippocampal function related to spontaneous activity using c-Fos immunoreactivity. c-Fos is commonly used as a marker of integrated electrical activity^33,34^. We euthanized paired WT and γ2* animals directly from their home cages, and immunostained frozen tissue sections for c-Fos immunoreactivity. Homozygous γ2* animals exhibited a reduced number of c-Fos+ cells in the granule cell layer of the dentate compared with matched WT animals (Fig. 6A). The intensity of labeled neurons in the dentate granule cell layer did not clearly differ between genotypes (22 ± 17% lower fluorescence in paired mutant animals). The weaker c-Fos activation results support the idea of abnormal hippocampal activity in a major input layer to the hippocampal circuit. Labeling was generally not evident in either genotype in the CA1 region, making comparison difficult. Also, we note that the technique does not distinguish between lower overall activity or more distributed activity at levels sub-threshold for activating c-Fos. Further, sensitivity of c-Fos may not be sufficient to detect subtle changes to activity.

We assessed changes to interneuron density by staining for somatostatin, which labels mainly dendrite-targeting interneurons and for parvalbumin, which labels mainly soma-targeting interneurons. We found no difference in the density of either cell type, suggesting that secondary changes to interneuron numbers are unlikely to explain the reduced c-Fos staining or the abnormal EEG (Fig. 6B,C). To test whether secondary changes to granule cell morphology might explain the differences in c-Fos immunoreactivity, we performed a Sholl analysis of granule neuron dendritic morphology. The mutant granule cells showed dendritic branching patterns comparable to that of WT cells (Fig. 6D).

**Figure 7:**
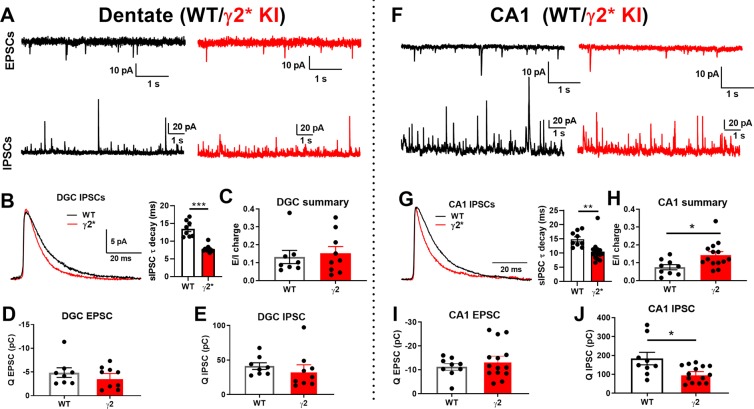
Excitation/Inhibition (E/I) ratio is modestly elevated in hippocampal CA1 neurons from γ* KI slices. (**A**) Representative traces of sEPSCs and sIPSCs from DGCs in WT vs γ2* KI slices. (**B**) Averaged waveform of sIPSCs from DGCs in representative WT and γ2* KI slices and pooled data, revealing the accelerated decay of γ2* IPSCs (P < ***1e-5). (**C**–**E**) Summary of E/I charge ratio, total charge transfer for EPSCs and IPSCs in WT vs γ2* KI DGCs (N = 8 WT cells from 2 animals and 9 γ2* KI cells from 2 animals, P = 0.708, 0.455 and 0.518, respectively; 2 tails independent t test). (**F**) Representative traces of sEPSCs and sIPSCs from CA1 cells in WT vs γ2* KI slices. (**G**) Average waveform of sIPSCs from representative CA1 cells in WT and γ2* KI slices, revealing the accelerated decay of γ2* IPSCs (P = **0.007). (**H**–**J**) Summary of E/I charge ratio, total charge trasfer for EPSCs and IPSCs from WT vs γ2* KI CA1 cells (N = 9 WT cells from 4 animals and 14 γ2* KI cells from 4 animals, P = *0.020, 0.361 and *0.014, respectively; 2 tails independent t test).

Given that the anatomical changes examined did not readily explain hippocampal functional differences, we turned to *ex vivo* slices to examine spontaneous synaptic activity in DGCs, the main input relay cells of the hippocampus, and in CA1 pyramidal neurons. Spontaneous IPSCs exhibited the accelerated decay expected from studies of recombinant receptors (Fig. 7A,B,F,G; Supplementary Figure S1). However, the total charge of sIPSCs was unaltered in DGCs and was only altered by an amount expected of the change of IPSC decay in CA1 pyramidal neurons (Fig. 7E,J; Supplementary Figure S1). sEPSCs were not altered detectably in either cell type (Fig. 7D,I; Supplementary Figure S1). Altered sIPSCs did not result from exclusion of γ2* from receptors or from substitution by another subunit because sIPSCs were mostly insensitive to PTX (100 μM), a concentration that abolished sIPSCs in WT slices^16^.

To survey acute and secondary impact of the γ2* subunit on synaptic properties of DGCs, we examined the excitation to inhibition ratio (E/I ratio) in DGCs by summing the postsynaptic charge transfer of EPSCs and IPSCs over 60 s at the respective reversal potentials in order to isolate excitation and inhibition (Fig. 7). Pilot experiments identified the reversal potential for pharmacologically isolated IPSCs to be near −60 mV under these conditions. We found no statistical difference in the E/I ratio in pooled WT and γ2* data for DGCs (Fig. 7C), although the direction of the mean IPSC charge (Q) and E/I ratio was altered as expected from the truncated IPSCs (Fig. 7D,E). The result suggests that the IPSC decay difference is sufficiently subtle to be masked by variability in sIPSC frequency and amplitude. The same analysis of E/I ratio and integrated excitation and inhibition values was performed on CA1 pyramidal neurons (Fig. 7F–J). In this case, the elevated E/I ratio reached a statistical threshold of  P < 0.05 (Fig. 7H) in the direction expected from the change in inhibition. Only inhibitory charge was detectably reduced, suggesting that accelerated γ2* IPSCs explain the difference in E/I ratio. Overall, the change to inhibition in DGCs and CA1 cells paralleled the effect size of the change to E/I ratio and thus likely drives the E/I balance difference in γ2* cells.

**Figure 8:**
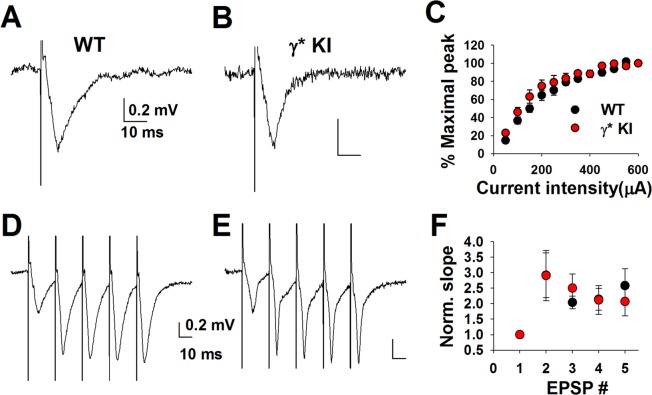
CA1 fEPSPs in response to a train of stimulus pulses are unaltered from γ* KI slices. (**A**,**B**) Representative traces of fEPSPs evoked in CA1 area from WT vs γ2* KI slices. (**C**) Input-output curve (peak amplitude vs stimulus intensity) of CA1 fEPSPs from WT (black) vs γ2* KI (red) slices. Genotype comparison: P = 0.211, two-way repeated measures ANOVA. (**D**,**E**) Representative traces of CA1 fEPSPs evoked by a 50 Hz, 5 pulses train from WT vs γ2* KI slices. (**F**) The 2^nd^–5^th^ fEPSP slope is normalized to the 1^st^ fEPSP slope following the 50 Hz, 5 pulse trains, from WT (black) vs γ2* KI (red) slices. N = 5 WT slices from 2 animals and 5 γ2* KI slices from 2 animals. Genotype comparison: P = 0.977, two-way repeated measures ANOVA.

We reasoned that the impact of altered inhibition may be more evident in the summed activity of many neurons, during trains of impulses, where both feedforward and feedback inhibition would participate. Surprisingly, we found no evidence that the input/output characteristics of dendritic CA1 fEPSPs evoked by stimulation in the Shaffer collateral pathway were impacted by γ2* (Fig. 8A–C). Decays of fEPSP waveforms were not demonstrably different, with decay time constants of 5.8 ± 0.8 and 4.9 ± 0.7 ms for WT and γ2* respectively (n = 6 each, P = 0.44). We further observed no change in the fEPSPs evoked by short 50 Hz trains (Fig. 8D–F). These results again suggest a mild impact of γ2* at the cellular and synaptic level in hippocampus.

**Figure 9:**
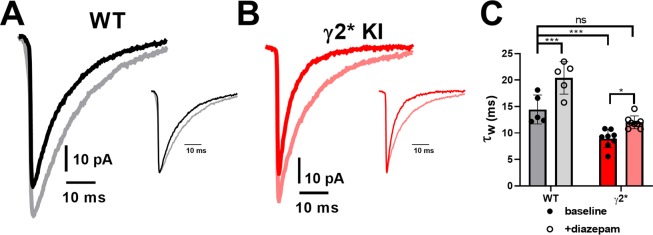
Diazepam rescues the γ2 sIPSC decay to near the WT level. (**A**) Representative traces of sIPSCs from WT vs γ2* KI DGCs, before (black) and after (red) 1 μM diazepam application. Insets showed normalized peak sIPSCs before and after diazepam for each genotype. (**B**) Summary of decay tau before and after 1 μM diazepam application, from DGCs in WT vs γ2* KI slices. N = 5 WT cells from 1 animal and 8 γ2* KI cells from 2 animals, P = ***0.001 for WT baseline vs WT diazepam; P = ***0.001 for WT baseline vs γ2* KI baseline; P = 0.226 for WT baseline vs γ2* KI diazepam; P = *0.032 for γ2* KI baseline vs γ2* KI diazepam; one-way ANOVA and Bonferroni’s multiple comparisons test.

If the impact of γ2* is mainly through the acute acceleration of IPSC deactivation, then we might expect to rescue the phenotype of γ2* animals by a pharmacological intervention that prolongs IPSCs, thereby restoring WT status. For this test, benzodiazepines are a good choice because they require the presence of a γ2 subunit, and our studies with recombinant receptors demonstrated that the mutant γ2 subunit retains benzodiazepine sensitivity (Fig. 3). To verify this in native cells, we recorded sIPSCs from DGCs in hippocampal slices (Fig. 9). Application of 1 μM diazepam prolonged both WT and γ2* IPSC decays as expected. The weighted time constant of γ2* IPSCs treated with diazepam was statistically indistinguishable from baseline WT IPSCs (Fig. 9B), suggesting that diazepam could be an effective pharmacological rescue strategy for the acute effects of the mutation.

**Figure 10:**
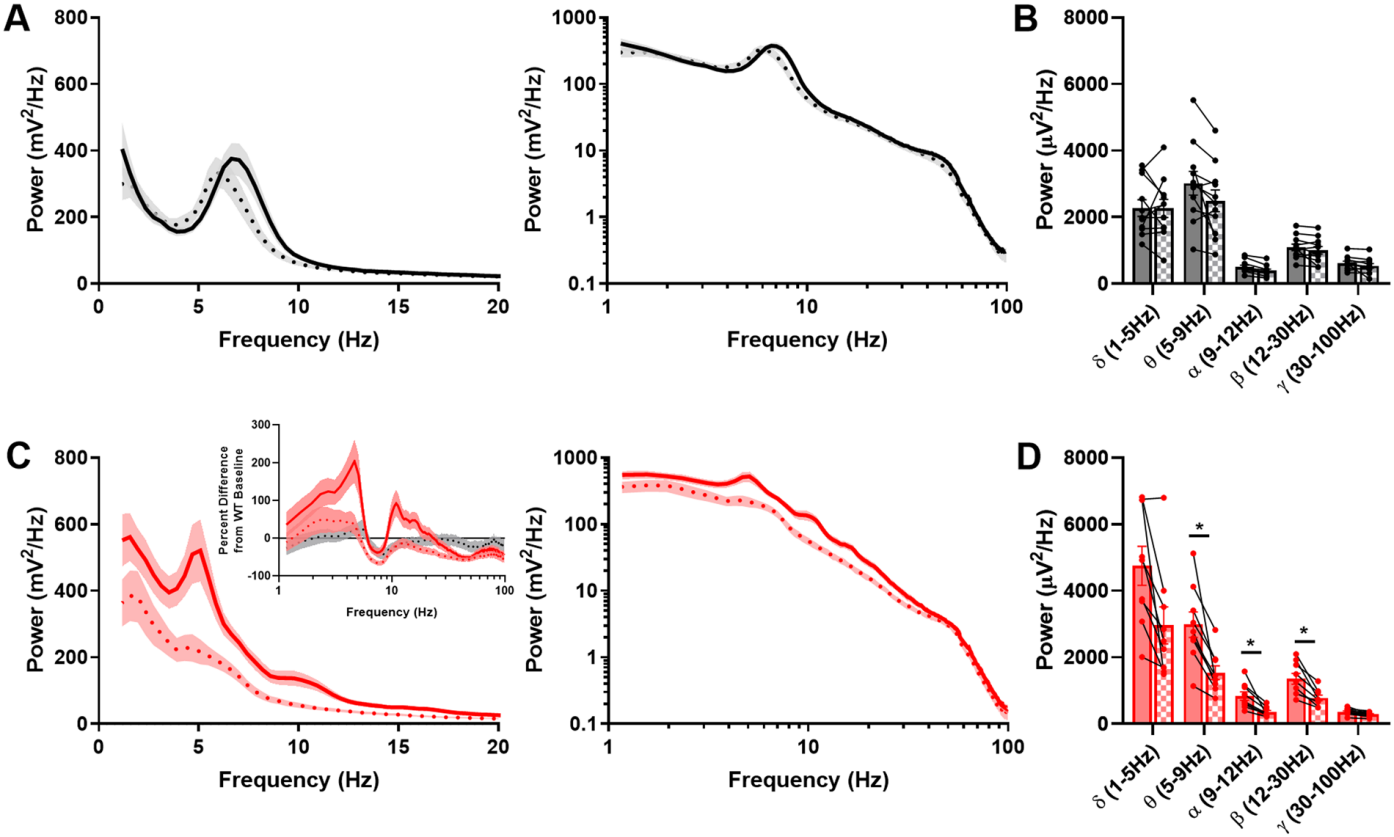
Diazepam reduces overall EEG power selectively in γ2* animals. (**A**) Active wake power spectra from WT animals (n = 11) before (solid line) and after (dashed line) non-sedative diazepam injections (0.75 mg/kg). (**B**) Effect of diazepam on power of indicated EEG bands in WT animals. (**C**) Active wake power spectra from γ2* animals (n = 9) before and after non-sedative diazepam injections, inset percent difference of active wake power spectra from WT baseline, of WT after diazepam (black dashed line), γ2* baseline (red solid line), and γ2* after diazepam (red dashed line). (**D**) Effect of diazepam on EEG band power in γ2* animals. The result of a three-way ANOVA showed a significant interaction among frequency, genotype, and drug (F, (4, 90) = 5.25, P = 7e-4) and between drug and genotype (F(1,90) = 27.79, P = 9.2e-7). Comparisons in (**B,D**) show the results of false-discovery adjusted t tests as described in the Methods.

We next injected mice with 0.75 mg/kg diazepam (i.m.), which proved in pilot observations of spontaneous locomotion to be just sub-sedative in both genotypes. Cortical EEG from bouts of active exploration showed no discernible impact of diazepam in WT mice (Fig. 10A,B). By contrast, γ2* EEG proved more pharmacologically malleable. The higher power of low frequencies observed in γ2* baseline EEG was mitigated by diazepam (Fig. 10C,D). On the other hand, diazepam did not clearly restore the theta peak to the 6–8 Hz range observed in WT animals. The restoration of γ2* EEG power toward the WT profile can be seen in a plot of the percent difference in power at each frequency for each genotype and drug condition, compared with the WT baseline condition (Fig. 10C, inset). The changes to EEG were not associated with differences in the behavioral sensitivity of the two genotypes to diazepam. Both groups had similar amounts of activity for an hour following diazepam injection, based on video ratings (WT: 32:32 ± 2:13; γ2*: 37:52 ± 4:29 of activity). We conclude that the γ2* EEG can be partly rescued by pharmacological restoration of IPSCs, but latent secondary changes not amenable to pharmacological rescue may also contribute.

## Discussion

Our results show that mice homozygous for a mutation in the non-obligatory γ2 subunit exhibit disrupted hippocampal function, measured by EEG and c-Fos immunoreactivity. The underpinnings of the disruptions appear mild at the cellular and micro-circuit level, but are magnified at the level of cortical EEG. Pharmacological rescue of the cellular phenotype partially restores the EEG. Overall, our results demonstrate that chronic, mild perturbation of inhibition has a large impact on hippocampal function, consistent with human mutations in GABA_A_ receptor subunits. The mouse model we developed here represents a tractable system for exploring the impact of a well-characterized mutation on inhibition’s role in development and function.

Although we presented biophysical differences in receptor function early in the presentation of results, in truth our initial strategy sought to follow a path laid out in the development of mice carrying benzodiazepine-resistant subunits^28^. The idea was to generate mice whose GABA function is intact but that resist antagonism by picrotoxin, in order to separate cellular contributions of receptors bearing different non-obligatory subunits^16^. Past evidence suggested that the T6′Y amino acid substitution has no effect on basal function^13^. However, the behavioral phenotypes of the resulting mice caused us to perform a more detailed accounting of biophysical parameters of the mutated receptors. We confirmed previous work that found no change in the steady-state properties of receptors containing γ2*^13^. However, non-steady-state kinetics were accelerated, including IPSC decays in mouse neurons and deactivation of recombinant receptors to brief GABA applications. The lack of change to sIPSC amplitude or frequency^16^ suggest that the kinetic change is largely responsible for driving the phenotypes. A lack of change to GABA EC_50_ with accelerated deactivation kinetics also characterize the human K328M epilepsy mutation^12^. More work will be needed to reconcile the lack of change in sustained responses with the change in deactivation kinetics in these mutations. One possibility is that an undetected decrease in activation rate, not evident at high [GABA], could balance the decreased deactivation to leave GABA EC_50_ unchanged.

Because the mutation disrupts excitatory/inhibitory balance in favor of excitation and seizures, it may be surprising that a marker of integrated neural activity, c-Fos immunoreactivity, showed fewer positive cells in mutant hippocampi. At least two considerations may help explain the paradox. First, animals were not euthanized during or immediately after seizures. Therefore, hippocampal c-Fos expression was not likely the direct result of seizure activity. Second, the decrease of integrated activity likely reflects complicated circuit effects of the mutation, possibly even extrinsic to the hippocampus.

In principle, the change to inhibition could directly account for functional abnormalities, but constitutively altered inhibition could also cause secondary compensatory or pathological changes, leading to alterations that are less amenable to acute rescue of receptor kinetics. Our results detected no changes to the structure of principal neurons of the dentate gyrus or to numbers of several interneuron classes. Further, acute pharmacological intervention with diazepam had stronger impact on the EEG of mutant animals than WT animals and partially reverted the EEG phenotype. Thus, a specific secondary change was not evident under baseline conditions that would obviously contribute to altered hippocampal theta rhythm. Physiological evidence of circuit behavior in hippocampus, assayed through sEPSCs and sIPSCs and through evoked fEPSPs revealed no evidence for secondary changes that impact excitability or synaptic transmission in hippocampus. On the other hand, we cannot exclude the possibility of contributing secondary consequences since the effect of diazepam was partial. Further, we previously detected a depression of mIPSC frequency in these animals, presumably representing a secondary effect^16^. Ultimately, genetic restoration of WT γ2 at different times and locations should yield insight into secondary vs. acute explanations for the EEG phenotype.

Regardless of whether changes leading to the EEG phenotype are direct or secondary, the paradox between small cellular changes (slight acceleration of IPSCs, minimally disrupted E/I ratio) and a large EEG and altered c-Fos phenotype deserves further exploration. Future work may gain additional insight through meso-level *in vivo* or *in vitro* analyses, such as local field potential recordings or photometry. Cell-type selective knock-ins could also in principle yield insight because interneuron-selective γ2 knockouts exhibit phenotypic similarities to the γ2* mice^35^. It seems likely that ensemble-level disruptions of coordinated activity are likely to underlie the γ2* phenotype.

The γ2* mutation may offer a tractable model of genetic epilepsies. At least 11 different mutations in γ2 subunits characterize human epilepsy-related syndromes^12^. The T6′Y mutation is particularly reminiscent of the human K328M mutation^12^. The pharmacological fingerprint offered by the T6′Y mutation offers experimental tractability not offered by human mutations. For instance, in human mutations of GABA_A_ receptor subunits, the incorporation of the mutant subunit cannot readily be experimentally verified. Picrotoxin insensitivity allows us to exclude trafficking and incorporation deficits as explanations for abnormal functioning.

In summary, we introduce a model for exploring how small changes to inhibition can lead to large abnormalities in brain functioning. The deficiencies incurred appear to lie at the meso-level of population dynamics, since cellular changes are minimal. The results highlight the key role that finely tuned inhibition plays in coordinated function of CNS function.

## Supplementary information

Figure S1

## Data Availability

The datasets generated and analyzed during the current study are available from the corresponding author on reasonable request.
