# Supplementary material for: Mild chronic perturbation of inhibition severely alters hippocampal function
Source: Sci Rep. 2019 Nov 11;9:16431. doi: 10.1038/s41598-019-52851-w (PMC6848214; doi:10.1038/s41598-019-52851-w)
Supplement: Supplementary file 1 — Figure S1 [file 41598_2019_52851_MOESM1_ESM.pdf]

Mild chronic perturbation of inhibition severely alters hippocampal function.

Min-Yu Sun, Luke Ziolkowski, Peter Lambert, Hong-Jin Shu, Micah Keiser, Nicholas Rensing, Natasha Warikoo, Monika Martinek, Carson Platnick, Ann Benz, John Bracamontes, Gustav Akk, Joe Henry Steinbach, Charles F. Zorumski, Michael Wong, Steven Mennerick

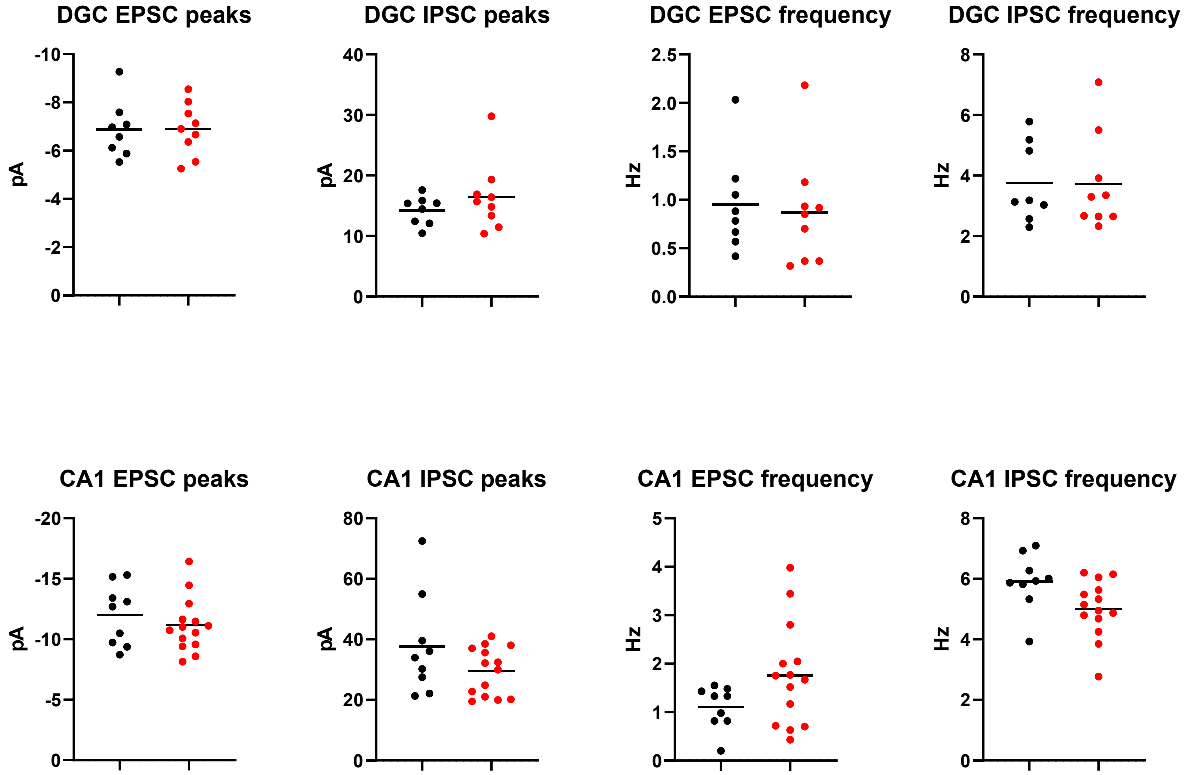

Supplementary Figure S1. sIPSC parameters from DGCs and CA1 recordings. The pooled data plots represent the data underlying Figure 7.
